# Pediatric Spinal Solitary Fibrous Tumor: A Systematic Review of a Rare Condition

**DOI:** 10.3390/children12091214

**Published:** 2025-09-10

**Authors:** Andrea Trezza, Chiara B. Rui, Stefano Chiaravalli, Veronica Biassoni, Elisabetta Schiavello, Sabina Vennarini, Ester Orlandi, Giorgio G. Carrabba, Maura Massimino, Carlo G. Giussani

**Affiliations:** 1 Neurosurgery, Fondazione IRCCS San Gerardo dei Tintori, Via Pergolesi 33, 20900 Monza, Italy; chiarabenedetta.rui@irccs-sangerardo.it (C.B.R.); giorgio.carrabba@unimib.it (G.G.C.); carlo.giussani@unimib.it (C.G.G.); 2 Pediatrics, Fondazione IRCCS Istituto Nazionale dei Tumori, 20133 Milan, Italy; stefano.chiaravalli@istitutotumori.mi.it (S.C.); veronica.biassoni@istitutotumori.mi.it (V.B.); elisabetta.schiavello@istitutotumori.mi.it (E.S.); maura.massimino@istitutotumori.mi.it (M.M.); 3 Pediatric Radiotherapy, Fondazione IRCCS Istituto Nazionale dei Tumori, 20133 Milan, Italy; sabina.vennarini@istitutotumori.mi.it; 4 Department of Clinical, Surgical, Diagnostic, and Pediatric Sciences, University of Pavia, 27100 Pavia, Italy; ester.orlandi@cnao.it; 5 Radiation Oncology Unit, Clinical Department, CNAO National Center for Oncological Hadrontherapy, 27100 Pavia, Italy; 6 School of Medicine and Surgery, University of Milano-Bicocca, 20126 Milan, Italy

**Keywords:** spinal tumor, solitary fibrous tumor, pediatric, pediatric neuro-oncology

## Abstract

**Highlights:**

**What are the main findings?**

- Pediatric SFTs are a rare tumor with only five cases reported in the literature, all of which show good functional outcomes and no recurrence at follow-up.
- Gross total resection remains of primary importance for the treatment of SFTs; adjuvant therapies (radio and/or chemotherapy) are lacking standardized pediatric protocols, with a predilection of radiotherapy if not contraindicated.

**What are the implication of the main findings?**

- Management of SFTs in the pediatric population must be tailored to each patient and surgery should be the first choice.
- The role of radiotherapy, chemotherapy and targeted agents in improving long-term disease control is still controversial.

**Abstract:**

**Background:** Spinal solitary fibrous tumors (SFTs) are a rare oncological entity, almost anecdotal in the pediatric population. They have a high relapse rate and represent an ongoing oncological challenge. **Methods:** In this article, we conducted a systematic review starting from a case report to highlight the current state of the art in managing these tumors. **Results:** Spinal solitary fibrous tumors (SFTs) are rare, slow-growing neoplasms that can be either intra- or extramedullary. Only a limited number of studies focus on primary pediatric spinal cord localization. Five pediatric cases of spinal SFT have been documented in the literature. On MRI, they typically present as highly vascularized, contrast-enhancing masses. Histologically, they are composed of spindle-shaped cells within a collagenous stroma featuring staghorn-shaped blood vessels. More aggressive subtypes, such as dedifferentiated SFTs, resemble high-grade sarcomas. The NAB2–STAT6 fusion is a key marker, driving EGFR signaling, collagen production, and fibrosis. Additional diagnostic markers include CD34, CD99, and Bcl-2. Surgical resection remains the primary treatment. In metastatic cases, chemotherapy—mainly with anthracyclines, dacarbazine, or temozolomide—is employed, although no standardized pediatric protocols exist. Anti-angiogenic agents, including tyrosine kinase inhibitors, have shown promise. Radiotherapy is used postoperatively for local disease control, but its impact on survival is still under investigation. **Conclusions:** Surgery remains the cornerstone of treatment, significantly impacting the natural history of the disease and symptom control. While clinical trials exploring radiotherapy and chemotherapy are ongoing in adults, no specific treatment protocol has been established for pediatric patients.

## 1. Introduction

Solitary fibrous tumors (SFTs) are a rare entity arising from mesenchymal cells, described for the first time by Klemperer and Rabin in 1931 [1]. Since the identification of the NAB2-STAT6 fusion gene in 2013, it has become a well-defined entity. SFTs are rare tumors with an incidence rate of 0.61 and 0.37 per million persons per year for extra-meningeal [2] and meningeal [3,4] cases, respectively. They can arise ubiquitously in the body with the most frequent localizations being pleura [5,6,7,8] and abdomen/pelvis [9,10,11,12]. Head [13,14,15,16] and neck [17] with a sinus or meningeal location [4,18] can be a presenting site as well, while more rarely SFTs can involve the spinal cord and its meningeal layers [19,20,21]. Due to their strong propensity for recurrence and metastatic progression, these tumors present an ongoing oncological challenge for which effective treatments remain limited to date [12,22,23].

Both in adult and pediatric populations, surgery is mandatory and a gross total resection seems to have a major impact on prognosis, allowing disease control and reducing the recurrence rate. Postoperative radiotherapy is recommended in order to lower the recurrence rate [24], and many studies on chemotherapy agents and immunotherapy are currently exploring alternative strategies, although the outcomes so far have been not fully satisfying [25,26,27,28,29]. The majority of clinical and molecular data on SFTs are described in small series of adult patients or mixed populations. Within case reports on SFT, pediatric spinal SFTs are an even more rare entity [21,30,31].

**Figure 1:**
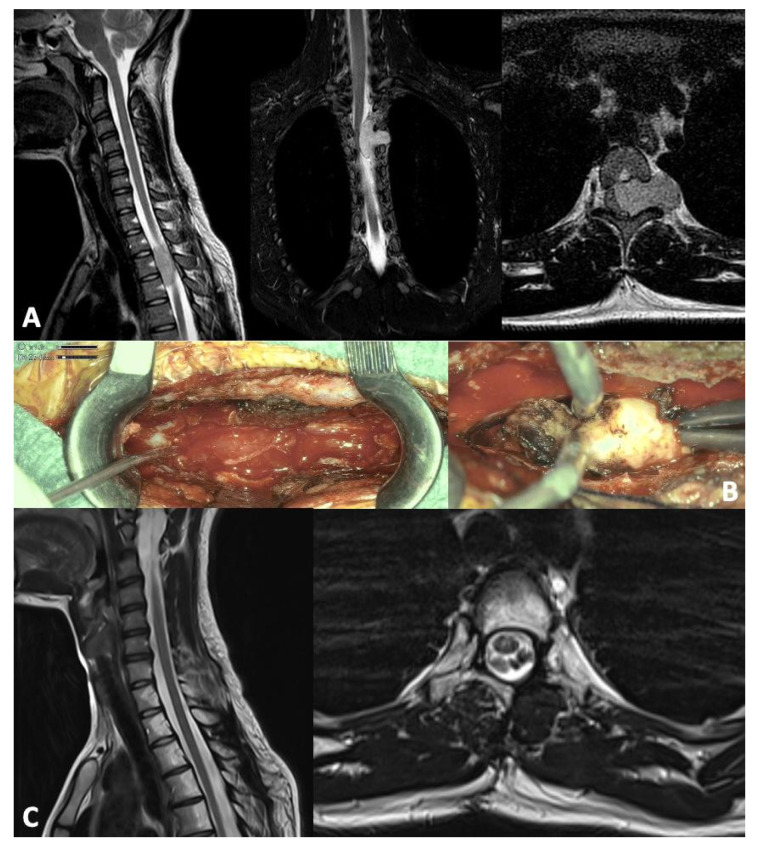
Preoperative T2-weighted MRI showing intradural extramedullary lesion with compression and dislocation of the spinal cord at level D3–D4 and left foraminal extension (**A**). Intraoperative views after left-sided laminectomy, appearance of solid fibrous mass at level D3–D4 with high blood supply (**C**). T2-weighted MRI at 2 years follow-up with no sign of relapse (**B**).

**Case report**. We report the case of a 17 year old patient, otherwise healthy, who presented at our institution for increasing interscapular pain unresponsive to analgesics, with a spinal MRI showing a dural lesion at D3–D4 level, conditioning cord compression, and mild signs of local myelopathy (Figure 1A). At the first clinical evaluation, the patient complained of a radicular pain in D3–4 involving the left arm and mild tetraparesis. Brain MRI was negative for other pathological findings.

A total microsurgical resection with a 3D-4K exoscope and intraoperative neuromonitoring (IONM) was performed through a left-side D3–4 hemilaminectomy (Figure 1B). At discharge, a complete remission of preoperative symptoms was evident. Postoperative MRI showed complete tumor resection.

Histological examination revealed a high cellularity lesion, composed of elongated cells with oval nuclei. Mitoses were rare, and no necrosis was observed.

Immunohistochemistry demonstrated positivity for STAT6 and CD34 (focal). EMA, GFAP, S100, SOX10 and Melan-a were negative. The histologic diagnosis concluded for a solitary fibrous tumor (grade 2), confirmed by the central pathological review.

**Figure 2:**
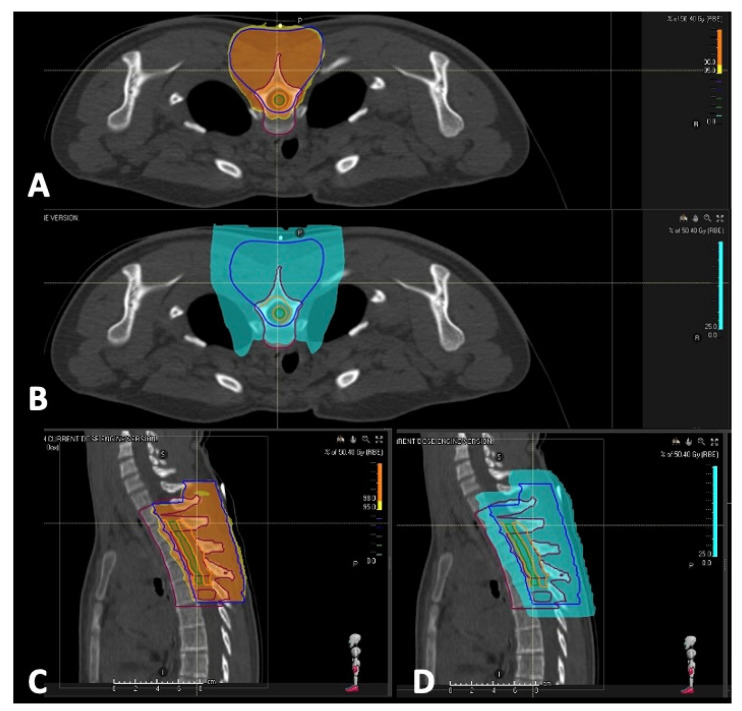
Axial (**A**) and sagittal (**C**) CT view showing target coverage at 98% of the prescription dose over the entire Clinical Target Volume (CTV). The two tumor volumes are, respectively, blue for the precautional CTV and orange for the Boost CTV. Note the dose gap (green) intended to spare the central portion of the spinal cord. (**B**,**D**) illustrate the dosimetric physical properties of the proton plan, with the distribution of low irradiation doses and the near-complete sparing of tissues and vital organs (esophagus, heart, lungs) anterior to the vertebral bodies.

Due to the site of the disease and to the gross-total surgical resection, radiotherapy was performed to consolidate the local treatment and to achieve disease control. This treatment decision was borrowed from recommendations of the European paediatric Soft tissue Sarcoma Study Group (EpSSG) [32]. In order to spare healthy tissues and reduce acute toxicities, proton therapy was chosen. The patient underwent proton therapy (Figure 2) with a total dose of 50.4 Gy Relative Biological Effectiveness (RBE) on the posterior aspect of dorsal spine (from T1 to T5), with a daily dose of 1.8 Gy (RBE) for 28 days with a boost on the initial volume of disease of 1.8 Gy (RBE) for fraction into two fractions up to a total of 30 days of therapy. Subsequently, a boost with a dose of 3.6 Gy (RBE) for a total dose of 54 Gy (RBE), 1.8 Gy per fraction, total number of fractions = 30, on the initial disease volume was administered.

No toxicities were reported during the treatment. The last MRI performed 2 years after the end of proton therapy showed no sign of relapse (Figure 1C).

Neurological examination is today characterized by hypoesthesia of the left scapular-dorsal region extending up to the anterior chest wall. Sporadic neuropathic pain in the left shoulder is reported but there is adequate pain control without medications.

Taking these reports as a starting point, we will resume the state of the art of this peculiar entity in the pediatric age.

## 2. Materials and Methods

**Figure 3:**
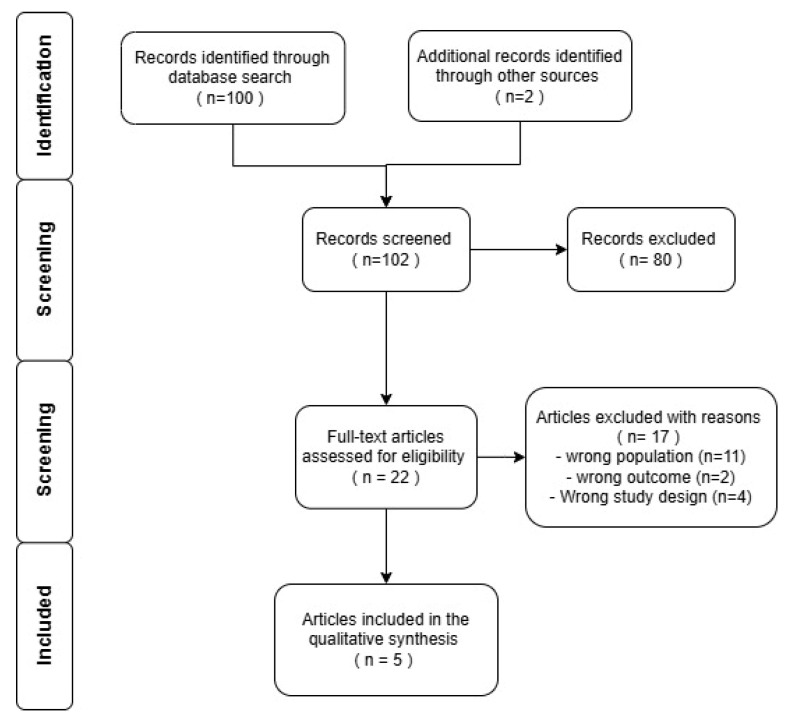
Prisma flow-chart. We selected and analyzed 22 out of 102 articles found in our literature investigation. Eleven articles were excluded because of population issues (e.g., exclusive adult patients, miscellaneous of tumors). Four articles were excluded because they were clinical trials for validation of new therapies in adult SFTs. At the end of this process, a total of 5 articles were considered for the purpose of the present review.

A systematic review of the literature and meta-analysis according to the Preferred Reporting Items for Systematic Reviews and Meta-Analyses (PRISMA) protocols were performed to define the state-of-the art for primitive spinal solitary fibrous tumors in children (Figure 3).

The review was conducted on the PubMed database, using the following string: (pediatric solitary fibrous tumor [MeSH Major Topic]) AND (solitary fibrous tumor [MeSH Terms]) AND (spinal tumor [MeSH Terms]) OR (intramedullary tumor [Filter]) OR (hemangiopericytoma [MeSH Terms]) AND (English [Filter]). All titles and abstracts were screened double-blinded in order to exclude irrelevant studies. Embase, Scopus, Web of Science, DOAJ, and gray literature sources were considered in the initial search strategy and included in the PRISMA flow-chart.

Due to the limited amount of literature available, all retrospective studies were considered, including reviews, cohort studies, cross-sectional studies, case series and case reports. Inclusion criteria were as follows: English language articles with full text available and published in the last 25 years, pediatric series, diagnosis of primitive spinal solitary fibrous tumor. Articles including mixed tumor population and other neoplastic localizations than spinal cord were excluded.

## 3. Results

A total of 102 articles were analyzed for the review. We selected and considered 22 out of 102 articles found in our literature investigation. Eleven of these 22 articles were excluded because of population issues (e.g., exclusive adult patients, miscellaneous of tumors). Four articles were excluded because they were clinical trials for validation of new therapies in adult SFTs. At the end of this process, a total of 5 articles were considered for the purpose of the present review [30,31,32,33,34] (Figure 3). Table 1 reports a qualitative analysis of these articles (Table 1).

A total of 5 pediatric patients with spinal SFT have been reported. Location was cervical in 2 cases, dorsal in 2 patients and in the last patient an extensive localization from C7 to the conus was described. Age at diagnosis ranged from 10 months to 18 years, four patients were males and one female. All tumors were intradural, three were extramedullary and two had an intramedullary invasion.

As far as adjuvant therapies were concerned, only Singla et al. [31] described a case of a 12-year-old female patient with intradural extramedullary D11-L1 STF treated with radiotherapy and adjuvant chemotherapy (ifosfamide and epirubicin) after gross-total surgical resection.

In all the other reports [29,31,33,34,35], treatment was limited to surgical management without any adjuvant oncological treatment [36,37].

## 4. Discussion

### 4.1. Radiological and Histological Characteristics

Spinal SFTs are rare slowly growing tumors which can be both intra- or extramedullary. In the study of Dauleac et al. [38], they are described as brightly contrast-enhanced in MRI due to the high vascularization and having hyposignal in T2 sequences. For patients with meningeal localization [19], a whole-body CT or MRI is mandatory in order to fully define the tumor as “solitary” [39]. Intraoperative features are varied and different, depending on the localization, the tumor consistency and the organ contiguity. It is well known and reported that surgery can be challenging due to dural, bone or neural tissue infiltration [40].

Morphologically, SFTs are composed of cells with spindle or ovoid shape within a collagenous stroma, with blood vessels in a staghorn shape, in a “patterless pattern” [41]. Histologically, they can be either paucicellular with abundant stroma or highly cellular tumors where collagen is hardly detected and have a low mitotic count [37,42,43]. Subtypes that are more aggressive are described as dedifferentiated SFTs, which exhibit a transition zone to high-grade sarcoma, including components of rhabdomyosarcoma or osteosarcoma with rare osseous and cartilaginous metaplasia [42].

### 4.2. Molecular Characterization

The identification of *NAB2–STAT6* was important in terms of understanding and treating this entity [37,43,44,45]. Since the discovery of this molecular feature, testing for the NAB2-STAT6 fusion and STAT6 nuclear expression has been recommended in order to classify the tumor as SFT [37,43,46]. NAB2-STAT6 fusion is considered a defining characteristic in the most recent WHO classification. The prognostic value of this molecular feature is still controversial and needs further research to be validated; more data about outcomes are available only for non-CNS localizations.

*NAB2-STAT6* fusion leads to a dysregulation of EGFR signaling with overexpression of EGFR1 [47,48]. NAB2-STAT6 is also involved in different pathways such as collagen production, fibroblast activation and vessels formation, and, ultimately, induction of fibrosis [49,50].

Other non-specific markers for the diagnosis of SFTs are CD34 and CD99. CD34 is a membrane glycoprotein present in mesenchymal stem cells and in more than 80% of SFTs; however, in aggressive STFs phenotypes it can be lost [51,52]. CD99 is largely expressed in SFTs and encodes for a transmembrane protein implicated in different functions like exocytosis, cell adhesion and differentiation [53]. This protein was proved to have a role in metastasis development; however, in SFTs, it is found more in early stages than in the advanced disease [54,55]. Bcl-2 is reported to be diffusely expressed in SFTs [51,56,57] as well as in synovial sarcoma or in physiological stem cells and endocrine tissue [58].

### 4.3. Oncological Management

Despite the extensive literature describing features and characteristics of adult patients with solitary fibrous tumor, only a few series report on primary spinal cord localizations and even fewer cases concern the pediatric age. Moreover, the need and the role of an adjuvant oncological treatment is still unclear, while surgical resection still remains the first-choice and most relevant treatment with a significant impact on the prognosis of these tumors [19,59,60].

Literature about the oncological management of spinal pediatric SFTs is anecdotical and only one study reports on the use of both chemotherapy and radiotherapy in a pediatric patient [31]. Surgery alone could provide a disease cure in almost 60% of adult patients, but, often due to the tumoral infiltrative pattern and the localization, complete resection is not feasible [7], thus increasing the risk of recurrence [12,22,23,61].

As commonly observed in other brain and spinal tumors, a total surgical resection that takes into account the management of all the perineural tissues leads to a lower recurrence rate and a longer overall survival [18,40].

Chemotherapy is used mostly for the treatment of adult patients with metastatic malignant SFTs, while no shared protocols are available for children. In general, the main evidences on cytotoxic drugs are on anthracyclines [26], with possible addition of ifosfamide. Dacarbazine and temozolomide recently showed some efficacy in SFTs, especially in combination with bevacizumab [28] or doxorubicin [62]. Phase II trials with eribulin are now ongoing for patients with advanced SFT [63,64].

Because of the strong correlation between the NAB2-STAT6 fusion and vascular markers [65], anti-angiogenic drugs are a therapeutic option for adult patients, such as tyrosin-kinase inhibitors like sunitinib [27,66,67,68,69] or axitinib [70,71,72], even in combination with anti-PD-1 (nivolumab) [67]. Clinical trials for new multitarget compounds are ongoing, such as targeting VEGFR, PDGFRβ, FGFR, and KIT. For refractory SFTs, doxorubicin containing regimens [73], dacarbazine [74] and trabectedin are valuable options [25,73,75]. SFTs, though rare, can present challenges in treatment, and recent findings highlight the evolving role of antiangiogenics in managing these tumors [41]; the use of these drugs has been shown to provide superior disease control. Recent research has shown that rotating antiangiogenic drugs can indeed be more effective for treating non-dedifferentiated SFTs as compared to single-agent chemotherapy. The synergy between chemotherapy and antiangiogenics has emerged as a promising treatment in prolonging overall survival. This combination approach works by addressing both the tumor’s direct growth and its ability to maintain itself through blood supply, thus enhancing overall outcome [41].

Currently, there are no published studies on the use of chemotherapy in children with STFs, while some studies are available in the adult population.

Postoperative radiotherapy is commonly used, especially in cases of tumors with anaplastic features, which are even rarer. It is suggested to lower the recurrence rate [40]. However, although SFTs seem to be sensitive to radiotherapy [76], its survival benefit is yet to be proved. For spinal and meningeal SFTs, adjuvant radiotherapy is recommended for both adults and pediatric patients to consolidate the local disease control, improving overall survival [77,78]. Local or whole-spine radiotherapy could be a useful instrument, especially for those cases with a high mitotic index or with residual tumor [76,77,78].

## 5. Conclusions

Spinal SFTs are a rare entity with few reports in the literature and even fewer on the pediatric population. Only a few pediatric cases can be found in the literature, all of which have good functional outcomes and no recurrence at follow up. Surgery appears to be the most important treatment in terms of disease control and progression-free survival rate. Lack of follow-up data and the small number of pediatric patients reported account for the lack of a shared and common treatment that can be necessarily tailored to each patient. Even as trials with radio/chemotherapy approaches are ongoing in the adult setting, no pediatric protocol has been evaluated yet. Considering the lack of available data about the pediatric population, our case of a single child with SFT is worthy of reporting.

## Figures and Tables

**Table 1:** Literature review with the most relevant case series described. Number of patients, symptoms at presentation, surgical entity of resection and limitations are reported.

Authors	Type of Study	Patients	Symptoms	Tumor	Treatment	Outcome	Limitations
Albert et al., 2017 [33]	Case report	1 (10 y.o./M)	Right upper extremity weakness	Intradural extramedullary C1–C3	GTR	Disease free	Only one pediatric patient
Singla et al. 2020 [31]	Case series	10 (1; 12 y.o./F)	Acute conus cauda syndrome for 3 days	Intradural extramedullary D11–L1	GTR + RT+ CT (epirubicin, ifosfamide)	Disease free	Heterogeneous population (9 adults, 1 child)
Tamburrini et al. 2003 [34]	Case report	1 (10 months old/M)	Fasciculation in right leg, styosis, hypertonia in legs	Intramedullary from C7 to the conus	Partial resection	Spontaneous regression of residual. Mild paraparesis	Early study, case report
Jallo et al. 2005 [35]	Case series	4 (1; 17 y.o./M)	Scoliosis and spastic paraparesis	Intramedullary D5–D6 (two lesions)	GTR	Disease free, mild scoliosis	Case report, limited FU (1.6 years)
Brunori et al. 1999 [30]	Case report	2 (1; 18 y.o./M)	Paresthesias, limb weakness, urinary retention	Intradural extramedullary from clivus to C3	GTR	Disease free at 12 months	Early study, limited FU
Trezza et al. 2025	Case report	1 (17 y.o./F)	Left interscapular unresponsive pain	Extramedullary intradural lesion D3–D4; mild myelopathy and cord compression	GTR + RT (protons; 50.4 Gy)	Disease free at 24 months	Case report

## Data Availability

No new data were created or analyzed in this study.
